# Arthroscopic treatment of early glenohumeral arthritis

**DOI:** 10.1007/s10195-012-0219-6

**Published:** 2012-11-22

**Authors:** Giuseppe Porcellini, Giovanni Merolla, Fabrizio Campi, Andrea Pellegrini, Chandra Sekhar Bodanki, Paolo Paladini

**Affiliations:** Unit of Shoulder and Elbow Surgery, D. Cervesi Hospital, Via L. Van Beethoven 1, CAP, 47841 Cattolica, RN Italy

**Keywords:** Early arthritis, Shoulder, Arthroscopy, Cartilage lesions

## Abstract

**Background:**

The articular cartilage of the shoulder is not endowed with intrinsic repair abilities, so the detection of chondral lesions during arthroscopy may indicate that additional articular procedures are needed. The aim of the current study was to evaluate the benefits of arthroscopy in patients with early shoulder arthritis, and to assess which clinical and radiological features are correlated with better arthroscopic outcomes.

**Materials and methods:**

Out of a total of 2,707 shoulders, 61 arthroscopies were performed on patients aged 30–55 years suffering from a painful early arthritic shoulder. We performed a retrospective study of 47 of those 61 patients with osteoarthritis at Samilson–Prieto stage I or II. SST and Constant score were used as outcome measures. Arthroscopic circumferential capsulotomy was performed to release the soft tissues and increase the joint space. Glenoid chondral lesions were caregorized according to location (anterior, posterior, centered) and size (small, large, total) and treated with microfractures; in the last 11 patients, we placed a engineered hyaluronic acid membrane, Hyalograft^®^ C, on the surface of the glenoid. Postoperative care included mobilization the day after surgery, with the arm protected in a sling for two weeks. Follow-up examinations were performed at 3, 6, 12, and 24 months after surgery. The clinical and radiographic data collected were compared with those obtained at the last examination.

**Results:**

The mean Constant score increased from 43.8 points to 79.1, and the mean SST score increased from 4.9 points to 9.4 points. Clinical outcomes improved significantly in 44 patients (93.6 %). The three patients (6.4 %) with the lowest scores showed progression of arthritis. Age, gender, glenohumeral distance, and presence of engineered hyaluronic acid membrane were not related to clinical scores. Recovery of range of motion as well as small and centered cartilage lesions were statistically associated with improved outcome.

**Conclusion:**

The main finding was that soft tissue procedures (including capsulotomy and synovectomy) associated with glenoid microfractures are only suitable for patients with early arthritis and preserved humeral head shape, particularly in cases with small and centered glenoid cartilage lesions.

## Introduction

The articular cartilage of the shoulder is not endowed with intrinsic repair abilities; therefore, when a disease such as instability or cuff injury is present, even minor lesions can rapidly lead to early glenohumeral joint arthritis. Cartilage lesions are not unusual, even in young patients [1], and are often found during arthroscopic procedures performed when such patients have various pathologic conditions [2–4]. Less common conditions include glenoid dysplasia and osteochondritis dissecans [5]. The varying thickness of joint cartilage and resistance properties of the subchondral bone [6] result in lesions with different depths and widths, depending on the resistance offered by the articular surface [7, 8]. Minor cartilage lesions associated with rotator cuff or glenohumeral ligament damage will induce topographically different stresses on the various areas of the articular surface. Recent and older research findings have shown a correlations between cartilage wear and lesion site and between site and symptoms in the shoulder as well as in the knee [9–12]. Several conservative options are available to manage shoulder arthritis: alleviate pain, reduce inflammation, and (especially) halt or at least slow down the evolution of arthritis [13]. Such therapies entail changes in lifestyle as well as systemic and topical drug administration. Viscosupplementation using hyaluronic acid may be a useful treatment option in patients who have shoulder osteoarthritis with an intact rotator cuff [14], while less satisfactory results have been obtained in those with rotator cuff tears or advanced osteoarthritis [15]. Several surgical options are available to manage primary shoulder arthritis, including simple arthroscopic joint debridement [16] and more complex techniques such as resurfacing using fascia lata or meniscus [17], osteochondral autologous transplantation [18], resurfacing arthroplasty [19], and total arthroplasty [20]. The use of microfractures to treat full-thickness chondral defects is a viable option that provides good results in young patients, with the greatest improvements seen for smaller lesions of the humerus and the worst results observed in patients with bipolar lesions [21], even when the microfracture is covered with a periosteal flap [22]. The microfracture technique enhances chondral resurfacing by providing a suitable environment for new tissue formation and taking advantage of the body’s own healing potential [23]. A combination of microfractures and viscosupplementation with three weekly injections of intraarticular hyaluronic acid was seen to have positive effects on the repair tissue that formed within the chondral defect at an early follow-up examination: it had possible chondroprotective and anti-inflammatory effects and limited the development of degenerative changes within the joint [24]. The use of an engineered hyaluronic acid membrane gave good results in pilot studies in the knee, whether using the scaffold alone or the scaffold loaded with autologous chondrocytes [25]. The aim of the current study was to evaluate the benefits of arthroscopy in patients with early shoulder arthritis and to assess which clinical and radiological features are correlated with better arthroscopic outcomes.

## Materials and methods

All patients gave informed consent prior to being included in the study. This was a retrospective study that was authorized by the local ethical committee and performed in accordance with the Ethical Standards of the 1964 Declaration of Helsinki as revised in 2000.

Out of a total of 2,707 shoulder procedures performed from January 2006 to December 2008, 61 (2.25 %) arthroscopies were performed on patients aged 30 to 55 years (mean 41.7) suffering from a painful early arthritic shoulder. The patients were males in 45 cases (73.8 %) and females in 16 cases (26.2 %). A single surgeon performed arthroscopic surgery using a similar arthroscopic technique in all patients. All patients had a preoperative imaging study with X-ray evaluation of the shoulders, leading to classification according to the Samilson and Prieto scheme [26]. Arthritis was grade I or II in all cases. An additional MRI was performed to image the cartilaginous defects on both surfaces. SST and Constant score were used as outcome measures [27, 28]. The treatment approach was selected on the basis of clinical history and imaging data.

Inclusion criteria were: arthritis at Samilson–Prieto stage I or II, passive stiffness <40° in forward flexion and <30° in external rotation with the arm at the side. During arthroscopy, cartilaginous defects of the glenoid were classified as small (<2 cm^2^), big (>2 cm^2^), or total (the defect covers the whole surface). All the glenoid cartilage defects were grade IV [29, 30] or ICRS grade 4a/b [11]. Arthroscopic examination of the humeral head showed that cartilage was still present and the humeral head shape had been maintained. Patients with broad and deep humeral cartilage defects and a squared head were excluded from the study. Of the 61 shoulders treated for painful early arthritis, 50 (82 %) met the inclusion criteria. Since 3 (6 %) patients were lost to follow-up, the study was conducted on 47 patients (94 %)—males in 35 cases (74.5 %) and females in 12 cases (25.5 %).

Exclusion criteria were passive shoulder stiffness with a loss of forward elevation of >40° and a loss of external rotation of >30°, previous surgery, nerve palsy, and rotator cuff tears.

### Radiographic evaluation

Preoperative X-ray imaging was used to calculate the distance between the glenoid and the humeral head surface.

Radiographic examination was executed as follows. An anteroposterior radiograph in neutral shoulder rotation with the patient standing, a scapular lateral (outlet) radiograph, and an axillary view were obtained at the final follow-up. The articular space was evaluated preoperatively and at the last follow-up by measuring the distance between the glenoid and the humeral head surface on the axillary radiographs [30]. All measurements were performed using OsiriX imaging software (v.3.7.1).

### Surgical technique

**Fig. 1:**
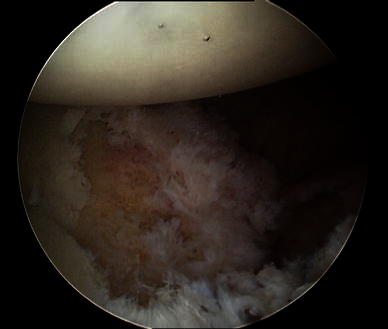
Glenoid surface after delineating the boundaries of the cartilage lesions

**Fig. 2:**
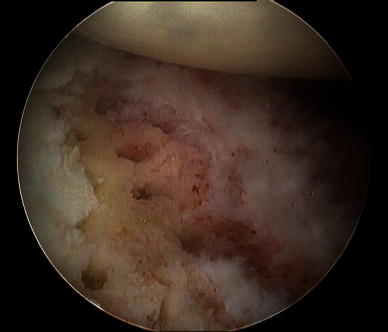
Glenoid surface after performing microfracture

**Fig. 3:**
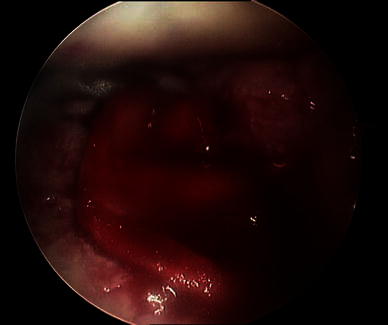
Engineered hyaluronic acid membrane lying on the glenoid surface without fluid irrigation and traction

Patients were placed in lateral decubitus with 5 kg of traction. Three routine arthroscopic portals (anterior–superior, anterior–inferior, and posterior) were used to perform the surgical technique. After initially removing the synovial membrane, a circumferential capsulotomy was performed to achieve release the soft tissues in order to increase the joint space. Rotator interval debridement with removal of hypertrophic synovitis was performed in all cases. Loose bodies were removed if present. After delineating the boundaries of the cartilage lesions (Fig. 1) or of the whole glenoid (leaving the glenoid labrum in situ) and debriding the calcified chondral layer (until punctate bleeding was observed), we implemented microfractures, placing the awl holes at appropriate positions perpendicular to the subchondral plate at 2–3 mm intervals [23] (Fig. 2). For the final 11 (23.4 %) patients, after performing the microfractures, we placed an engineered hyaluronic acid membrane (Hyalofast^®^, Fidia Advanced Biopolymers S.r.l., Abano Terme, Italy) on the glenoid surface. The membrane was first cut into the shape of the glenoid chondral lesion and placed without using fixation devices such as screws or fibrin glue. The placement of the membrane was achieved by passing it through a 8.5 mm cannula in the anterior–inferior portal. A global inspection of the joint, without fluid irrigation and traction (Fig. 3), was performed at the end of the procedure in order to evaluate the stability of the membrane during humeral head movement.

### Postoperative rehabilitation

The rehabilitation program simply involved the use of a sling for the first two weeks after surgery. Immediate passive mobilization began the day after the operation, under the supervision of a physiotherapist. Pool exercises and active assisted exercises within the pain-free range of motion were started three weeks after surgery. Active exercises to balance the internal and external rotators of the shoulder with a rubber band were initiated after eight weeks. Additionally, for all patients, physical therapy was performed in our institution’s outpatient rehabilitation unit for about six months to strengthen the shoulder and maximize the range of motion until maximum improvement was achieved.

Follow-up examinations were done (as per the usual routine) at 3, 6, 12, and 24 months from surgery. The collected data were compared to those from the last examination. X-ray and final clinical examinations were performed at the two-year follow-up. The radiographic classification of arthritis developed by Samilson and Prieto [26] was used to follow arthritic changes in the shoulder from preoperative to final follow-up radiographs.

### Statistical methods

Statistical analysis was performed with the Intercooled Stata 9.0 software package for Windows (Stata Corporation, College Station, TX, USA). A logistic regression model was developed to investigate the influences of the selected factors on outcome score (dependent variable). Variables were eligible for incorporation into the model if they were significantly (*p* < 0.05) associated with a positive trend in the outcome. The variables examined as potential predictors (independent factors) were patient age, gender, pre- and postoperative loss of forward elevation, pre- and postoperative loss of external rotation with the arm at the side, pre- and postoperative distance between the glenoid and the humeral head on the axillary X-ray, type of glenoid cartilage lesion [small (<2 cm^2^), large (>2 cm^2^), or total (all of the surface of the glenoid), position of the glenoid cartilage lesion (anterior, posterior, or center), use of an engineered hyaluronic acid membrane. These variables were considered to be dichotomous (value: 0/1). The relationship between each factor and increase in outcome score was tested with the *χ*^2^ test (bivariate analysis). The Pearson correlation coefficient (PCC) was employed to assess the interobserver reliability for outcome and arthritis classification, as evaluated by three different observers.

## Results

The mean Constant score of the two groups at the time of operation was 43.8 points (SD 12.9). At two years of follow-up, the mean Constant score had reached 79.1 points (SD 14.9; *p* < 0.05) (Table 1).

**Table 1:** Pre- and postoperative constant scores at the final follow-up

Patient	Preoperative constant score	Postoperative constant score	Increase in score
1	52	74	22
2	48	85	37
3	61	84	23
4	31	75	44
5	38	80	42
6	41	84	43
7	24	79	55
8	46	75	29
9	54	89	35
10	32	81	49
11	60	75	15
12	31	78	47
13	38	86	48
14	43	70	27
15	36	68	32
16	38	74	36
17	47	78	31
18	28	91	63
19	36	79	43
20	38	84	46
21	41	86	45
22	45	73	28
23	57	61	4
24	60	88	28
25	58	87	29
26	31	68	37
27	46	85	39
28	53	83	30
29	41	89	48
30	49	78	29
31	55	52	−3
32	45	70	25
33	35	86	51
34	69	79	10
35	56	88	32
36	37	83	46
37	29	72	43
38	37	41	4
39	36	85	49
40	41	79	38
41	55	84	29
42	57	89	32
43	42	85	43
44	31	77	46
45	20	86	66
46	47	86	39
47	65	89	24
Mean	43.8	79.1	35.3
SD	12.9	14.9	15.1
Max	69	91	66
Min	20	41	−3

The mean SST score changed from 4.9 points (SD 1.8) to 9.4 points (SD 1.9) (Table 2). The PCC was close to 1 (0.9143), indicating the low variability in the outcome measurement.

**Table 2:** Pre- and postoperative SST scores at the final follow-up

Patients	Preoperative SST	Postoperative SST	Increase in SST
1	8	9	1
2	4	11	7
3	3	12	9
4	6	10	4
5	5	9	4
6	4	7	3
7	5	8	3
8	6	9	3
9	7	10	3
10	3	11	8
11	4	9	5
12	5	12	7
13	5	9	4
14	6	8	2
15	3	9	6
16	7	11	4
17	6	9	3
18	5	10	5
19	6	12	6
20	7	12	5
21	4	10	6
22	3	11	8
23	2	3	1
24	4	8	4
25	5	9	4
26	4	11	7
27	6	10	4
28	4	10	6
29	7	7	0
30	5	8	3
31	5	5	0
32	3	8	5
33	3	10	7
34	8	11	3
35	3	9	6
36	4	8	4
37	5	9	4
38	2	3	1
39	6	11	5
40	5	10	5
41	6	9	3
42	7	8	1
43	2	9	7
44	4	8	4
45	7	9	2
46	8	8	0
47	5	8	3
Mean	4.9	9.1	4.1
SD	1	2.3	2.3
Max	8	12	9
Min	2	3	0

There were no statistical differences among the three different observers in the PCC analysis (*p* < 0.05) of the outcome scores.

Twenty-one patients (87.5 %) had good outcomes. Three patients (12.5 %) had poor outcomes that were related to the progression of arthritis to Samilson–Prieto III and a squared humeral head (patient nos. 23, 31, and 38).

No statistical differences were found at X-ray examination between the pre- and postoperative glenohumeral distances: it remained a mean of 2.4 mm (range 1–4 mm, SD 1.60) (*p* > 0.05) (Table 3).

**Table 3:** Variate analysis (relationships of variables to increases in the Constant and SST scores)

Variable	*p* (*χ*^2^ test)
Patient age	0.91
Male	0.83
Female	0.86
Increase in forward elevation	0.04
Increase in external rotation	0.03
Glenohumeral distance	0.91
Glenoid cartilage lesion type	
Small	0.03
Large	0.49
Total	0.75
Position of glenoid cartilage lesion	
Anterior	0.16
Posterior	0.25
Centered	0.01
Use of engineered hyaluronic acid membrane	0.54

Age and sex were not related to outcome (*p* > 0.05; Table 3).

When the results were stratified, we found that small (<2 cm^2^) and centered glenoid lesions (*p* < 0.05) gave better clinical scores, while treatment with an engineered hyaluronic acid membrane had no affect on the final outcome (*p* > 0.05); (Table 3). Patients with involvement of the whole glenoid surface had the poorest outcomes (*p* > 0.05; Table 3).

## Discussion

Arthroscopy allows joint irrigation with removal of cartilage debris, cytokines, and inflammatory mediators [10]. Arthroscopic debidement associated with capsular release may provide significant pain relief and improve ROM in patents with capsular contracture of >15° [31]. Just as other research findings have shown how osteochondral lesions of >2 cm^2^ are correlated with persistent pain as a predictive variable for the ultimate failure of the arthroscopic procedure [31], the patients with the worst outcomes in our study were those with large cartilaginous defects. Severe arthritis does not seem to be usefully treated with arthroscopy because of deteriorating outcomes over time and poor functional results [32, 33]. Shoulder arthritis is followed by progressive restriction of ROM due to the contracture of the capsule and deformity of the humeral head [16]. For a peripheral cartilage lesion, the restriction of the glenohumeral joint volume, the compression of the damaged glenoid cartilage surface, the pivot mechanism, and the eccentric loads can all promote squaring of the humeral head [12]. 360° capsulotomy reduces compression between the humeral head and the glenoid and can therefore lead to an improvement in the ROM. This procedure is mandatory in all cases involving an arthroscopic approach to stiff arthritic joints. Patients affected by degenerative joint diseases with residual joint space can improve shoulder function and obtain pain relief after arthroscopic debridement. The unchanged glenohumeral distance indicates that the arthritic process is stable, and biological resurfacing of the glenoid with an engineered hyaluronic acid membrane does not appear to lead to better outcomes than debridement and capsulotomy.

In a young, active person with a focal symptomatic chondral lesion, arthroscopic approach with capsulotomy, debridement, and microfractures could be a plausible option to achieve a good outcome and (probably) delay arthritic evolution. In cases with large lesions, the arthroscopic approach appears to give fair outcomes and a deterioration over time. The effects of arthroscopic debridement in cases of degenerative shoulder disease have been explored by Van Thiel et al. [32], who reported favorable results on pain relief and recovery of shoulder function in 55 out of 81 selected patients at an average follow-up of 27 months, even if there are some notable differences between our study and that of van Thiel et al. [32], such as a lower grade of arthritis.

Who are the best candidates for arthroscopy in shoulder osteoarthritis? Based on the results of the current study, young men aged 30–55 years old with a small, centered glenoid cartilage lesion and a mild loss of ROM should benefit from this treatment. Data collected in this study cannot guarantee a certain perspective in patients arthroscopically managed for early shoulder osteoarthritis. We need more long-term follow-up data, a large case series, and a histological evaluation of second-look cases before considering the procedures described in this paper as reliable and safe.

The study has numerous limitations: (1) the lack of a control group; (2) various kinds of articular lesion were treated; (3) the lack of postoperative MRI control group; (4) the lack of an arthroscopic second look allowing the histological analysis of the soft tissue, which could resolve the issue of the difference between the normal fibrocartilage created after implementing microfractures and the features of the fibrocartilage grown on a scaffold of hyaluronic acid membrane. Given the aforementioned limitations, the main findings of this work are that progression of symptomatic arthritis was seen in only 12.5 % of the patients (three cases), and that the soft tissue procedures (capsulectomy and synovectomy) associated with microfractures are suitable for use in this type of patient. Arthroscopic capsular release delays disease progression by reducing load forces and improving ROM and joint elasticity.
